# Influence of heparin molecular size on the induction of C- terminal unfolding in β2-microglobulin

**Published:** 2016-12

**Authors:** Kanon Fukasawa, Yuichiro Higashimoto, Yoshihiro Motomiya, Yoshinori Uji, Yukio Ando

**Affiliations:** 1 Department of Chemistry, Kurume University School of Medicine, Kurume, Fukuoka, Japan; 2 Suiyukai Clinic, Kashihara, Nara, Japan; 3 Department of Medical Technology and Sciences, School of Health Sciences at ukuoka, International University of Health and Welfare, Okawa, Fukuoka, Japan; 4 Department of Neurology, Graduate School of Medical Sciences, Kumamoto University, Honjo, Kumamoto, Japan

**Keywords:** β2-microglobulin, Heparin, Dialysis-related amyloidosis, Biolayer interferometry

## Abstract

Dialysis-related amyloidosis (DRA) is characterized by accumulation of amyloid β2- microglobulin (β2m) in the interstitial matrix. Matrix substances such as heparin have reportedly been strongly implicated in the pathogenesis of dialysis-related amyloidosis. In clinical setting of hemodialysis, two types of heparin, i.e., high and low molecular heparin (H.M.H. and L.M.H.) have been routinely used. Still commonly used is H.M.H., followed by L.M.H. preparations with distinct advantages. Here, we studied that the interaction of native and two amyloidogenic β2m variants: ΔN6β2m and D76N β2m with H.M.H. and L.M.H. We also investigated whether heparin could induce β2m to have an amyloidogenic conformation. Biolayer interferometry revealed that ΔN6β2m had a strong reaction and D76N β2m had a moderate reaction with H.M.H.. Furthermore,H.M.H. induced the C-terminal unfolding in a native β2m. By contrast, L.M.H. showed no reaction even with ΔN6β2m. This study showed firstly a direct binding of β2m with H.M.H.. H.M.H. would provoked a C-terminal unfolding of β2m, which indicated production of an amyloidogenic intermediate, i.e., β2m92-99. In addition, our findings also suggest that L.M.H. may provide beneficial effects against the development of the DRA.

## INTRODUCTION

Since carpal tunnel syndrome was first reported in hemodialysis (HD) patients, why connective tissue was so often involved in dialysis-related amyloidosis (DRA) has been of particular interest in the investigation of the pathogenesis of this amyloidosis [1-5]. Connective tissue consists of collagen I, hyaluronate, and several kinds of glucosaminoglycans (GAGs) with or without SO3− groups. Among GAGs with SO3− groups in the human body, heparin is an essential molecule and is known to contain many SO3− groups. Two types of heparin, i.e., high molecular heparin (H.M.H.) with M.W > 10.0 K dalton, and low molecular heparin (L.M.H.) with M.W < 10.0 K dalton, have been commonly used as anticoagulant in the clinical setting of HD [6].

On the other hand, as is also well known, β2-microglobulin (β2m) is a precursor protein in DRA [7]. In 1997, Stoppini et al had reported that the monoclonal antibody specific for the C terminal region of β2m could inhibit an amyloid formation *in vitro *[8]. Subsequently, we demonstrated a C-terminal unfolded β2m in amyloid tissue from HD patients [9]. We thus believed that the C-terminal unfolding must be a critical conformational change in the transition from the native β2m to the amyloidogenic β2m.

Furthermore, we determined that the C terminus from 92Ile to 99Met unfolded completely in an amyloidogenic variant, i.e., ΔN6β2m, which lacked the six N-terminal amino acids [10].

Recently, Valleix et al. reported the first naturally occurring structural variant of β2m, Asp76Asn β2m (D76N β2m), discovered in members of a French family who developed progressive bowel dysfunction with extensive visceral β2m amyloid deposits despite normal renal function and normal circulating β2m concentrations and with none of the osteoarticular deposits characteristic of dialysis-related amyloidosis [11].

In this study, therefore, we analyzed the binding of native β2m with heparin and compared it with that of two amyloidogenic β2m variants: ΔN6β2m and D76N β2m. We then investigated whether heparin at a clinical dosage could induce C-terminal unfolding. We also suggested that serum β2m concentrations of 2.0 μM might be set as the target level before HD (i.e., the pre-HD serum β2m concentration) in HD patients.

## MATERIALS AND METHODS

**β** **2** **m and heparin: **Purified β2m, which was used as the native β2m, and two kinds of heparin, i.e., H.M.H.(>15,000 M.W.), and L.M.H.(4000-6000 M.W.) were purchased from Sigma (St. Louis, MO, USA).

**ΔN6β** **2** **m and Asp76Asn β** **2** **m (D76N β** **2** **m): **As previously reported, ΔN6β2m and Asp76Asn β2m (D76N β2m) were produced at the genetic engineering laboratory of Hokkaido System Science Co., Ltd. (Sapporo, Japan) [10].

**mAb92-99: **A monoclonal antibody specific for the C terminus of β2m (mAb92-99) was produced, as reported earlier [9].

**Biolayer interferometry (BLI) analysis** ***: ***BLI binding experiments were conducted at room temperature with a BLItz instrument (ForteBio, Menlo Park, CA, USA) [12]. Briefly, biotinylated heparin was immobilized on a streptavidin biosensor and subjected to 10 min of rehydration in the reaction buffer (phosphate-buffered saline with 0.09% Tween-20) before carrying out the binding experiments. The immobilization of the biotinylated heparin to the sensor was performed with 4 μL of 0.5 U/mL biotinylated heparin in the drop holder for 120 s followed by a 120 s incubation of the sensor in the reaction buffer. The binding reaction occurred in 4 μL drops containing various concentrations of the three species of β2m with agitation. The Kd value was obtained by fitting the data via the Data Analysis Software (ForteBio).

## RESULTS

**Figure 1:**
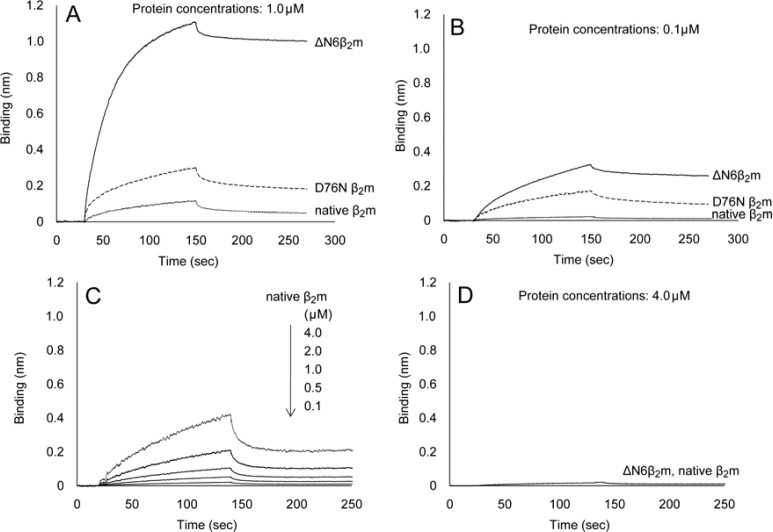
Analysis of the interaction of β2m variants with heparin

Binding of the native β2m, ΔN6β2m, and D76N β2m with heparin was studied at 0.1 and 1.0 μM by means of BLI. ΔN6β2m showed a strong reaction and D76N β2m showed a moderate reaction with H.M.H. (Fig. 1A). The BLI response level at 0.1 μM ΔN6β2m was 0.3 nm (Fig. 1B), which corresponded to that for 1.0 μM D76N β2m. However, the native β2m showed only a slight reaction at 1.0 μM and a questionable but possible reaction at 0.1 μM (Fig. 1A, B). The Kd values of native β2m, ΔN6β2m, and D76N β2m to H.M.H. were 3.71 x 10-6 M, 2.07 x 10-8 M, and 1.72 x 10-7 M, respectively (Fig. 1A, B). We also analyzed the interaction between native β2m and H.M.H. at various native β2m concentrations (4.0, 2.0, 1.0, 0.5, and 0.1 μM). BLI response levels of native β2m with H.M.H. clearly depended on the native β2m concentrations (Fig. 1C). The BLI response level at the 1.0 μM native β2m concentration was nearly 0.1 nm. By contrast,

L.M.H. showed no reaction even with ΔN6β2m (Fig. 1D).

**Figure 2:**
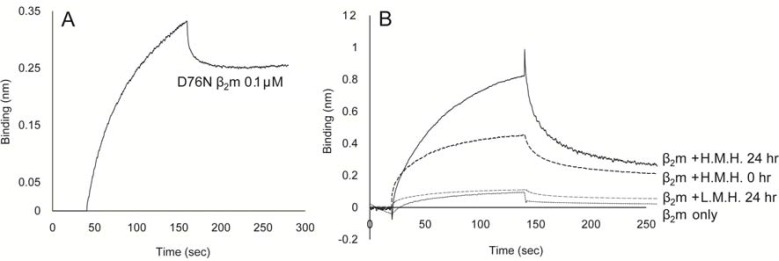
Analysis of the interaction of D76N β2m and native β2m in the presence of H.M.H. with mAb92-99

We next investigate whether the C terminus of D76N β2m unfolded. BLI at 0.1 μM D76N β2m clearly showed binding with mAb92-99, which indicated the C-terminal unfolding of D76N β2m (Fig. 2A). The Kd value of D76N β2m to mAb92-99 was 1.41 x 10-8 M. Native β2m was incubated with H.M.H. for 24 h, after which we used BLI with mAb92-99 to identify the β2m92-99 formed (Fig. 2B). We found a dose-dependent conversion to β2m92-99. On the other hand, L.M.H. did not induced c-terminal unfolding in native β2-microglobulin even after incubation for 24hr.

## DISCUSSION

Recent basic studies demonstrated that SO3− groups associated with GAGs are strongly implicated in β2m amyloidogenesis and that the interstitial matrix is a site that contains amyloidogenic β2m, which contributes to development of DRA *in vivo *[13, 14]. Borysik et al. reported that ΔN6β2m interacted in a time-dependent manner with heparin (sigma, H8537) to form amyloid fibrils under neutral conditions [15]. ΔN6β2m was often found in amyloid tissues in HD patients and is believed to be produced via proteolysis in the interstitial space [16, 17]. Therefore, suppression of interactions with GAGs should be important for preventing development of DRA.

Biotinylated H.M.H. (0.5 U/mL) was immobilized onto streptavidin biosensor. Protein concentrations were 1.0 µM (A) and 0.1 µM (B), respectively. ΔN6β2m (line, ──), D76N β2m (dashed line, ----) and native β2m (dotted line,……). (C) Biotinylated H.M.H. (0.5 U/mL) was immobilized onto streptavidin biosensor and native β2m (4.0 µM,

2.0 µM, 1.0 µM, 0.5 µM, and 0.1 µM, from top to bottom) was used as the binding partner. (D) Biotinylated L.M.H. (5.0 U/mL) was immobilized onto streptavidin biosensor. ΔN6β2m (line, ──) and native β2m (dotted line, ……) were used as the binding partner. Protein concentration was 4.0 µM. Three- independent experiments were performed, respectively.

 mAb92-99 (10 µg/mL) was immobilized onto protein A biosensor and D76N β2m (1.0 µM) was used a as the binding partner (A). After adding native β2m (1.0 µM) to the reaction drop holder in the presence or absence of H.M.H. (0.5 U/mL), the real-time binding was monitored. Native β2m incubated with H.M.H and L.M.H. for 24 h was also used as the binding partner (B). Three-independent experiments were performed.

We previously reported that an amyloidogenic intermediate β2m, i.e., β2m92-99, similar to ΔN6β2m, may also be formed via interactions with GAGs in the extravascular space [18].

This study not only confirmed the binding of two amyloidogenic β2m variants, i.e., ΔN6β2m and D76N β2m, with H.M.H. but also suggested the binding potential of native β2m with H.M.H. (Fig. 1). In addition, this study demonstrated a clear difference in the intensity of binding between H.M.H. and the two β2m variants, ΔN6β2m and D76N β2m. Valleix et al., in 2012, first reported the amyloidogenicity of D76N β2m, as a natural amyloidogenic mutant. However, patients with D76N β2m showed no signs of chronic kidney disease, and their serum β2m levels remained near normal, i.e., about 0.1 μM or so [11]. ΔN6β2m demonstrated a moderate response even at 0.1 μM with H.M.H. (Fig. 1B), comparable to a response of D76N β2m, at 1.0 μM (Fig. 1A), which indicated a 10-fold difference in BLI response intensity between these two variants. A similar difference in binding affinity with collagen had reported between a ΔN6β2m and a native β2m [19]. Because the C terminus of D76N β2m was also confirmed as unfolded (Fig. 2A), the same as that of ΔN6β2m, a difference in BLI response levels between the two amyloidogenic variants may be due to a difference in the populations of molecules having C-terminal unfolding, that is, molecules with C-terminal unfolding should occur much more commonly in ΔN6β2m than in D76N β2m. A clinical report by Valliex et al. indicated no involvement of the skeletal system at normal serum levels of D76N β2m in their patients [11]. The BLI response level at normal serum levels, i.e., 0.1 μM D76N β2m, was 0.2 nm in this study (Fig. 1B).

Whereas, a dose-dependent study with native β2m that was incubated with H.M.H. showed the BLI response value to be equal to 0.2 nm at 2.0 μM (Fig. 1C). Given that this value of the BLI response indicates that the skeletal system would be safe from deposition of amyloid β2m, we may refer to 2.0 μM (0.2 nm of BLI intensity) as the target value of serum β2m concentration before HD.

We are not certain about the true *in vivo *concentrations of GAGs with SO3− groups in HD patients, but the heparin concentration of 0.5 unit/ml used in this study may be comparable to serum concentrations in HD patients undergoing systemic heparinization. Relini et al. [17] previously reported heparin strongly enhances the formation of β2- microgloblin amyloid fibrils in the presence of type I collagen. The nucleation kinetic theory proposed by Naiki et al. has been known as a basic model for amyloid fibril formation, which consists of two steps, i.e., nucleation and polymerization [20]. Bonysik et al. [15] showed that GAGs with SO3− groups inhibited depolymerization and stabilized amyloid fibrils, but our study here demonstrated that heparin directly generated an amyloidogenic β2m, i.e., β2m92-99, which was likely to form oligomers and lead to amyloid nuclei.

Heparin has been implicated also in other amyloidosis—in Alzheimer’s disease and systemic amyloidosis associated with serum amyloid A protein [21]. Ariga et al. recently reported that L.M.H. can reverse the process of amyloidosis; inhibit fibril formation by blocking the formation of β-plated structure [22]. A possible therapeutic approach using L.M.H. to interfere with the interaction between proteoglycans and amyloid β proteins and to arrest or prevent amyloidosis is suggested. We thus believe that heparin may have an important role in the unique clinical setting of HD patients undergoing systemic heparinization. Moreover, although the half-life of L.M.H. is prolonged compared with H.M.H., a single bolus injection may not be sufficient for patients dialyzing >4 hr. Davenport reported a single bolus dose of L.M.H. was adequate for >98% patients dialyzing for up to 6h [23]. Our result indicates that L.M.H. did not induce native β2-microglobulin to c-terminal unfolded protein even after incubation of 24hr. Our findings suggest that L.M.H. may provide beneficial effects against the development of the DRA.

Finally, this study might demonstrated definitely clinical advantage of L.M.H. compared with H.M.H. for prevention of development of DRA. An underlying mechanism by which L.M.H. give rise to less interaction with β2m might be due to few contents of SO3− group.

## References

[1] Odell RA, Slowwiaczek P, Moran JE, Schindhelm K (1991). Beta2-microglobulin kinetics in end-stage renal failure. Kidney Int.

[2] Floege J, Schäffer J, Koch KM (2001). Scintigraphic methods to detect beta2-microglobulin associated amyloidosis (A beta2-microglobulin amyloidosis). Nephrol Dial Transplant.

[3] Garbar C, Jadoul M, Noël H, van Ypersele de Strihou C (1999). Histological characteristics of sternoclavicular beta2-microglobulin amyloidosis and clues for its histogenesis. Kidney Int.

[4] Inoue S, Kuroiwa M, Ohashi K, Hara M, Kisilevsky R (1997). Ultrastructural organization of hemodialysis-associated beta2-microglobulin amyloid fibrils. Kidney Int.

[5] Jadoul M, Garbar C, Noël H, Sennesael J, Vanholder R, Bernaert P, Rorive G, Hanique G, van Ypersele de Strihou C (1997). Histological prevalence of beta2- microglobulin amyloidosis in hemodialysis: a prospective post-mortem study. Kidney Int.

[6] Lim W, Cook DJ, Crowther MA (2004). Safety and efficacy of low molecular weight heparins for hemodialysis in patients with end-stage renal failure: a meta-analysis of randomized trials. J Am Soc Nephrol.

[7] Gejyo F, Odani S, Yamada T, Honma N, Saito H, Suzuki Y, Nakagawa Y, Kobayashi H, Maruyama Y, Hirasawa Y (1986). β2-microglobulin: A new form of amyloid protein associated with chronic hemodialysis. Kidney Int.

[8] Stopping M, Bellotti V, Mangione P, Merlini G, Ferri G (1997). Use of anti-(beta2 microglobulin) mAb to study formation of amyloid fibrils. Eur J Biochem.

[9] Motomiya Y, Ando Y, Haraoka K, Sun X, Morita H, Amano I, Uchimura T, Maruyama I (2005). Studies on unfolded beta2-microglobulin at C-terminal in dialysis- related amyloidosis. Kidney Int.

[10] Motomiya Y, Higashimoto Y, Uji Y, Suenaga G, Ando Y (2015). C-terminal unfolding of an amyloidogenic β2-microglobulin fragment: ΔN6β2-microglobulin. Amyloid.

[11] Valleix S, Gillmore JD, Bridoux F, Mangione PP, Dogan A, Nedelec B, Boimard M, Touchard G, Goujon JM, Lacombe C, Lozeron P, Adams D, Lacroix C, Maisonobe T, Planté-Bordeneuve V, Vrana JA, Theis JD, Giorgetti S, Porcari R, Ricagno S, Bolognesi M, Stoppini M, Delpech M, Pepys MB, Hawkins PN, Bellotti V (2012). Hereditary systemic amyloidosis due to Asp76Asn variant β2-microglobulin. N Engl J Med.

[12] Concepcion J1, Witte K, Wartchow C, Choo S, Yao D, Persson H, Wei J, Li P, Heidecker B, Ma W, Varma R, Zhao LS, Perillat D, Carricato G, Recknor M, Du K, Ho H, Ellis T, Gamez J, Howes M, Phi-Wilson J, Lockard S, Zuk R, Tan H (2009). Label- free detection of biomolecular interactions using BioLayer interferometry for kinetic characterization. Comb Chem High Throughput Screen.

[13] Ohashi K, Kisilevsky R, Yanagishita M (2002). Affinity binding of glycosaminoglycans with beta(2)-microglobulin. Nephron.

[14] Yamamoto S, Yamaguchi I, Hasegawa K, Tsutsumi S, Goto Y, Gejyo F, Naiki H (2004). Glycosaminoglycans enhance the trifluoroethanol-induced extension of beta2- microglobulin-related amyloid fibrils at a neutral pH. J Am Soc Nephrol.

[15] Borysik AJ, Morten IJ, Radford SE, Hewitt EW (2007). Specific glycosaminoglycans promote unseeded amyloid formation from beta2-microglobulin under physiological conditions. Kidney Int.

[16] Linke RP, Hampl H, Lobeck H, Hewitt EW (1989). Lysine-specific cleavage of beta2- microglobulin in amyloid deposits associated with hemodialysis. Kidney Int.

[17] Relini A, De Stefano S, Torrassa S, Cavalleri O, Rolandi R, Gliozzi A, Giorgetti S, Raimondi S, Marchese L, Verga L, Rossi A, Stoppini M, Bellotti V (2008). Heparin strongly enhances the formation of beta2-microglobulin amyloid fibrils in the presence of type I collagen. J Biol Chem.

[18] Motomiya Y, Uji Y, Ando Y (2012). Capillary electrophoretic profile of β2-microglobulin intermediate associated with hemodialysis. Ther Apher Dial.

[19] Giorgetti S, Rossi A, Mangione P, Raimondi S, Marini S, Stoppini M, Corazza A, Viglino P, Esposito G, Cetta G, Merlini G, Bellotti V (2005). Beta2-microglobulin isoforms display a heterogeneous affinity for type I collagen. Protein Sci.

[20] Naiki H, Hashimoto N, Suzuki S, Kimura H, Nakakuki K, Gejyo F (1997). Establishment of a kinetic model of dialysis-related amyloid fibril extension in vitro. Amyloid.

[21] Dudas B, Rose M, Cornelli U, Pavlovich A, Hanin I (2008). Neuroprotective properties of glycosaminoglycans: potential treatment for neurodegenerative disorders. Neurodegener Dis.

[22] Ariga T, Miyatake T, Yu RK (2010). Role of proteoglycans and glycosaminoglycans in the pathogenesis of Alzheimer’s disease and related disorders: amyloidogenesis and therapeutic strategies- A review. J Neurosci Res.

[23] Davenport A (2009). Review article: Low-molecular-weight heparin as an alternative anticoagulant to unfractionated heparin for routine outpatient haemodialysis treatments. Nephrology.

